# Medical Cases at the Buffalo Hospital of the Sisters of Charity

**Published:** 1849-05

**Authors:** Austin Flint

**Affiliations:** Attending Physician


ART. VII.—*Medical Cases at the Buffalo Hospital of the Sisters of Charity.* By Austin Flint, M. D., Attending Physician.

We purpose, under this head, to continue, in successive numbers of this Journal, reports of cases occurring at the *Buffalo Hospital of the Sisters of Charity*, selecting those which shall appear to possess special interest or importance. We select for this number, a single case.

*Case of circumscribed arachnitis, situated at the base of cerebellum, attended by effusion, terminating fatally, complicated with miliary tuberculosis of the lungs.*

Margaret O'Mier, aged 11 years, entered the Hospital, Jan. 22d., 1848. *History and Present Symptoms.*—Has suffered from cough, for the last two or three months. During a portion of this time, the cough was quite troublesome, being almost constant. Now, coughs but little. Has expectorated freely, but now the expectoration is slight. States that her previous health was good. Presents on neck, a cicatrix, but without the characteristic marks of a scrofulous cicatrix. She appears quite intelligent. States that she suffers from a sense of chilliness, much of the time, but has not had distinct chills, or rigors. Has had considerable pain at inferior part of sternum, of a dull character. Has also had stitch-like pains. Says she does not feel weak. Appetite is good. Bowels regular. Tongue lightly furred. Skin cool. Pulse 104. Says she has lost considerable weight. On physical examination of chest, the results were negative.

The following was prescribed:—S. ferri grs. ss., S. Quinine gr. 1, Sulph. acid aromat, gtt. viii three times daily.

*March 7th.*—Since former date, the patient has experienced, several times, lancinating pains in chest, continuing for two or three days, which have apparently been relieved by sinapisms. With this exception, she was able to be about, and to do light housework.

On physical examination on this date, a difference in *pitch of resonance*, was observed between the two sides in infra clavicular regions, and a *slight sibilant rale* in right infra clavicular region. Otherwise, no morbid signs discovered. The cough and expectoration, since her entrance, had been slight, almost insignificant. From the signs just noted, however, taken in connection with the stitch-like pains, and history, the case was regarded as one of incipient tuberculosis, and on this date, was so enrolled in the book of Hospital records.

*March 12th.*—The patient was in bed this morning, complaining of pain in head, and in left chest, also of weakness. These symptoms, on this day, were not regarded as calling for special treatment.

14th.—Continues to complain of headache, and remains in bed. Sinapisms to nuchæ.

15th.—Headache continues. Blister to nuchæ. Up to this time, there had been no febrile movement, and nothing to awaken suspicion as to any immediate danger.

16th.—A striking change in the character of the case. Pain in head continues. *Respirations sixty-two in a minute, and irregular. Pulse eighty, and irregular.* Physical examination revealed no new pulmonary affection, nor any cardiac affection. Skin was cool. Intelligence perfect.

Says she slept well. Manifests no unusual susceptibility to light or sounds. Muscular strength good. Bowels moved yesterday afternoon. Passes urine freely. She is disposed to sleep. Says she has slight nausea. No appetite. Tongue coated. Blister to nuchæ repeated, which had not properly vesicated, and calomel, grs. ii, prescribed, to be repeated at intervals, which, by a defect in the records, is not stated.

At evening, respirations very irregular, short, and catching. Pulse 72, very irregular and feeble. *Does not experience any suffering from embarrassment of respiration.* Has perspired freely this afternoon. Has less pain in head. Has slept considerably. Intelligence perfect.

Cont. med., and give, in addition, carb. ammonia, grs. ij., every two hours. Employ strongly sinapised foot bath, every four hours, and, for diet, a little essence of beef.

From the above assemblage of symptoms, it was evident that some serious cerebral trouble existed. That the affection was not one of extensive, or acute inflammation, was obvious from the absence of febrile movement, and other symptoms. That the cerebral hemispheres were not involved, was apparent from the fact that the intellect was perfect, the senses unaffected, etc. In short, the grave symptoms being limited to the respiration, and action of the heart, it was concluded that the affection involved particularly, and exclusively, the medulla oblongata. This opinion was stated in the clinical remarks on the case to the medical class then in attendance, being predicated upon the facts just referred to. The pains, it should be mentioned, were referred by the patient, to the occiput chiefly, With reference to the character of the cerebral affection, there did not appear to be any facts warranting a deffinite opinion. The particular portion of the encephalic structures involved, or immediately inflicted, was sufficiently clear, but the nature of the morbid condition was uncertain. Tubercular meningitis was suspected, from the fact that tuberculosis of the lungs was believed to exist, together with the evidences in the case of a scrofulous habit.

17th.—Reports better. Has still some pain in head, but less than yesterday. Respirations twenty, inspiration quick. Pulse eighty, skin warm. Continued treatment.

18th.—Continues better. No pain. Pulse sixty-eight, more regular. Respirations twenty, regular and natural. She is inclined to sleep, and fell asleep during the visit, but is easily roused. Bowels moved once yesterday. Skin warm. Tongue coated and moist. Has some appetite, and relishes the essence of beef.

Omit mercurial. Give iodide of potash, grs. iii, every four hours. Give the ammonia if respiration becomes irregular. Continue pediluvia.

19th.—Complains of some pain in the head this morning, greater in one part but not limited to that region. Speaks with embarrassment. Some apparent difficulty in expressing her ideas in words. Skin cool. Pulse 68. Respirations 28, sputa somewhat colored. Tongue coated and moist. Slight frowning, noticed for the first time. Bowels moved yesterday. Iodid. Potas. grs. x, every 4 hours. Let the hair be cut short, and apply blisters behind ears. *Evening.*—Slight frowning. Respriation improved. Pulse same. Pain in head continues, but is less than in morning. Has taken food with relish.

20th.—Reports better. No pain in head. Tongue cleaning. Says she was unable to sleep during the night, but was not kept awake by pain, or any distress. Respiration tranquil. Skin warm. Pulse normal. Appetite good. No dejection yesterday. On examination of spine, considerable tenderness was found to exist over the cervical and dorsal vertebræ. Accelerated and labored respiration was produced by pressure over these portions of the column.

In view of the latter symptoms it was hoped, and considered possible, that the affection might be functional, representing one of the protean forms called *spinal irritation.*

21st.—Suffered much during the night from severe pains over the whole body, as she states, coming on in paroxysms. Was unable to sleep. Feels better this morning. Has desire for food, but less than yesterday. No dejection yesterday. Respiration tranquil at first, but during the examination it became accelerated and labored. Pulse 100. Morphine had been given in night to relieve pain, and sinapisms applied to spine. Tongue coated and moist. Skin warm. Pressure over spine produces acute pain, and accelerated breathing. *She states that she sees objects double.* Apply blister over dorsal spine. Ol. Ricini ℥iij. Morphine to relieve paroxysm of pain, gr. $\frac{1}{8}$, *pro renata.*

*Evening.*—Respiration accelerated, and expiration accompanied by a groan. Double vision continues. Paroxysms of pain continue.

22d.—*Evening*—Suffered extremely from general pains during night, most severe in back. Respiration variable. Has slept much of the day, and tranquilly. Has bled from the nose during the night and day several times. Upper lip swelled. Tongue moist, with thick brown coating, except at tip, which is furred. Pulse 132. Skin warm and dry. No dejection for several days. Enema of infusion of Senna, to be repeated if free evacuation does not follow.

23d.—Sleeping tranquilly at the time of visit. Respiration natural. On being awakened, states that she is no better. Feels pain in head and back, and very weak. States that she had no better night than before, but she seemed to others to be more comfortable. Bowels moved freely last evening. Says she is hungry. Ate this morning bread and tea with relish. No nausea. Pulse 140. Double vision continues. There is slight strabismus. Paroxysms of pain continue to recur. Pulv. Doveri grs. ii, calomel grs. i, every four hours. Hoffman's anodyne, ʒi, *pro re-nata.*

*Evening.*—Has suffered much through day from pain in the back, head and extremeties. Pulse 128. Respiration irregular. Double vision continues. Continue med., adding sptts. terebinth., ʒss, in emulsion, every four hours, and increasing anodyne if pain is severe.

24th.—Died at 11, P. M.

Autopsy, 12 hours after death. Body presented moderate emaciation.

*Head.*—Moderate congestion, otherwise no morbid appearances of brain, except at inferior surface of cerebellum, over a space of about the size of half a dollar. Over this space, the arachnoid was opaque, somewhat thickened by lymph, and easily detected. Considerable effusion at base of skull, and fluid escaped freely from spinal canal. The effused fluid was sanguinolent, probably from being mixed with blood from vessels divided in removing the brain. Quantity of fluid estimated to be between 3 and 4 ounces. Lateral ventricles contained, each, about half an ounce of serum, slightly turbid.

The spinal marrow was examined as low as the fifth dorsal vertebra, and nothing morbid discovered.

*Chest.*—Tender adhesions of pleural surfaces, generally on both sides, over some portions quite recent. The upper portion of lungs, on each side, studded throughout with miliary tubercles. No tuberculous deposits larger than millet seeds, and no cavities. *Heart.*—Nothing abnormal.

*Abdomen.*—Nothing abnormal observed, except moderate enlargement of mesenteric glands. Stomach and intestines not opened. No lumbricoid entosoa in intestines, but two were partially protruded from anus. Intestines empty, and collapsed.

*Remarks.*—We may regard the sub-acute arachnitis, existing over a small, circumscribed space, situated just posteriorly to the medulla oblongata, as the pathological condition to which the result, and antecedent phenomena of this case are to be ascribed. The inflammation was so limited in extent, and of so little acuteness, that the early symptoms gave no intimations of the character of the disease, or the danger of the patient.

The development of the symptoms noted on the 16th of March, five days after headache was first experienced, marked the occurrence of effusion, sufficient to disturb, by compression, the functions of the medulla. The combination of symptoms then exhibited, indicated, in the first place, serious encephalic trouble. These symptoms were, irregular and greatly accelerated respirations, in connection with diminished frequency of the pulse; coolness of the surface; continued pain in head, none marked in occiput; and the absence of any evidences of a new pulmonary affection. It is to be observed, that notwithstanding the respirations were so frequent that the patient appeared to be panting for breath, no sense of embarrassment, or dyspnœa, was experienced. This denoted that the cerebral cause was so situated, as to impair the instinctive sense connected with the respiratory function. The fact that the phenomena of a grave cerebral affection, were almost exclusively manifested in the respiration, together with the absence of symptoms appertaining to inflammation, or other disease affecting the cerebrum, was sufficient to warrant the conclusion, that the disease was situated so as to involve the medulla oblongata, the direct relation of the latter to the respiratory function, being considered. Hence the diagnosis, in so far as the situation of the affection was concerned, the correctness of which, was verified by the autopsy. The same amount of arachnitis, elsewhere situated, for example, on one of the lateral surfaces of the cerebral hemispheres, would not have led to such a combination of symptoms—characterized by so disproportionate disturbance of the respiration; and situated, as it was, it was probably not sufficient to involve imminent danger, except from the effusion which occurred. Our attention has been directed, by several cases that have come under observation, to the occurrence of effusion at the base of the brain, and we are satisfied that it is a not unfrequent cause of death, occurring unexpectedly, and more or less uddenly, especially in children. Every observing practitioner is aware that disproportionate disturbance of the respiration, irrespective of pulmonary disease, is generally of bad omen, in whatever pathological connections it may be found. It is so, for the same reason that it was significant in this case, denoting encephalic trouble, involving the medulla oblongata, leading, in fatal cases, to asphyxia, by destroying the physiological sense presiding over the involuntary movements, necessary to the respiratory function.

The case is also one of interest with reference to the tuberculosis of the lungs. The fact of such extensive pleuritic adhesions so early in the progress of the tuberculosis disease is deserving of note, and serves to confirm the diagnostic value of the stitch-like pains, which are to be enumerated among the evidences of incipient Phthisis. The existence of miliary tubercles, without sufficient consolidation to produce decided dullness on percussion, and other marked signs, but nevertheless consisting with signs, slight in degree, but significant in character, upon which the diagnosis of tuberculous disease was based, may serve to illustrate the possibility of discriminating this affection even at so early a period. In this point of view the case was highly valuable, inasmuch as it is extremely rare that an opportunity is afforded of determining by autopsical examination the exact condition of the lungs in connection with the physical evidences of incipient tuberculosis.

April 20th, 1849.

